# Dynamic dual regulation of amphiregulin in liver pathophysiology: balancing regeneration and disease progression via the EGFR axis

**DOI:** 10.3389/fmed.2026.1697954

**Published:** 2026-02-18

**Authors:** Yi-Li Wang, De-Jiang Zhou, Qiong-Wan Wang, Ye Zhang, Wen-Jing Yang, Wen-Jie Tan, Hao-Yu Wang, Xiao-Wan Chen, San-Qiang Li

**Affiliations:** 1 The First Affiliated Hospital, and College of Clinical Medicine of Henan University of Science and Technology, Luoyang, Henan, China; 2 The Molecular Medicine Key Laboratory of Liver Injury and Repair, College of Basic Medicine and Forensic Medicine, Henan University of Science and Technology, Luoyang, Henan, China; 3 North Henan Medical University, Xinxiang, Henan, China; 4 Henan Center for Engineering and Technology Research on Prevention and Treatment of Liver Diseases, Luoyang, Henan, China; 5 Luoyang Key Laboratory of Transplantation and Immunological Studies for Haematological Diseases, Department of Clinical Laboratory, The First Affiliated Hospital, and College of Clinical Medicine of Henan University of Science and Technology, Luoyang, Henan, China

**Keywords:** amphiregulin, epidermal growth factor, liver, cancer, liver fibrosis

## Abstract

Liver diseases, ranging from acute injury to chronic fibrosis and hepatocellular carcinoma (HCC), represent a major global health challenge with limited therapeutic options. The liver’s inherent regenerative capacity can be disrupted during chronic disease, resulting in poor outcomes. However, this process is complex, as key regulatory molecules can exert opposite effects. A notable example is Amphiregulin (AREG), an epidermal growth factor receptor (EGFR) ligand whose role changes from promoting protective regeneration in acute injury to driving harmful processes in chronic settings. This review explores the dual and dynamic roles of AREG across the spectrum of liver diseases, with a focus on its expression patterns, downstream molecular pathways, and signaling networks that determine its functional transition from regeneration to fibrosis and carcinogenesis. These insights provide new theoretical foundations and potential intervention strategies for targeting the AREG-EGFR axis in liver disease therapeutics.

## 1 Introduction

The human endocrine system regulates physiological processes through the release of various regulatory factors, which are bioactive molecules with diverse structures and functions. These molecules from complex regulatory networks that control critical biological processes, including cell proliferation, inhibition of apoptosis, regulation of differentiation and migration, and cell cycle progression. The Epidermal Growth Factor (EGF) family is a key component of this network and includes major ligands, such as EGF, transforming growth factor-*α* (TGF-α), heparin-binding EGF-like growth factor (HB-EGF), betacellulin (BTC), epiregulin (EREG), and amphiregulin (AREG). These ligands exert their biological effects by binding specifically to EG receptors (EGFRs) on the cell membrane surface (1). While all EGFR ligands promote proliferation, AREG is distinct due to its: ① Early-response, damage-inducible nature (vs. constitutive ligands like EGF); ② Key role as a mediator between immune/inflammatory signals and epithelial repair; ③ Pronounced “double-edged sword” behavior across disease contexts, which is less pronounced for other ligands like HB-EGF or TGF-*α*. AREG was first identified by Shoyab et al. (2). Its name reflects its distinctive dual activity as it stimulates the proliferation of various epithelial cancer cells, such as melanoma cells, while inhibiting the growth of others, including A431 human epidermoid carcinoma cells. As a member of the EGF family, AREG functions primarily through the EGFR signaling pathway. It is highly expressed in the mammary ducts of adolescent animals, where it promotes duct morphogenesis and lactation maturation by inducing matrix metalloproteinases (MMP2/MMP9). Paradoxically, early characterization of this molecule also revealed potent growth-inhibitory effects on breast carcinoma cells. This contrast between physiological and pathological contexts highlights the complex molecular mechanisms underlying AREG’s functional versatility, and current translational research is focused on elucidating its dynamic regulation and context-dependent actions within tissue-specific microenvironments (Table 1).

**Table 1:** Comparative characteristics of EGFR ligands in the liver.

Ligand	Primary source cells	Primary functions	Effective concentration
AREG	Hepatocytes, liver cancer cells, Treg cells, cholangiocytes, HSCs	Liver regeneration, pro-fibrogenic effect, metabolic regulation, and hepatocarcinogenesis	High concentration
HB-EGF	Liver sinusoidal endothelial cells and hepatocytes	Liver regeneration and hepatoprotection	Low concentration
TGF-α	Hepatocytes, cholangiocytes	Hepatocyte proliferation and hepatocarcinogenesis	Low concentration
BTC	Cholangiocytes	Biliary regeneration	Low concentration
EREG	Hepatocytes, cholangiocytes, HSCs liver cancer cells (HCC, CCA), and immune cells (e.g., macrophages)	Liver regeneration, pro-fibrogenic, tumor promotion & progression, immune modulation	Low concentration

AREG originated from a genetic duplication event in the 4q13.3 region of the chromosome and subsequently diverged from ancestral EGF family genes. Following duplication, AREG retained the EGF-like domain but evolved distinct regulatory sequences and functional domains, thereby acquiring unique dual-function characteristics (3). Phylogenetic analyses indicate that AREG and HB-EGF share a common ancestral gene, with duplication occurring after mammalian divergence (4). This evolutionary trajectory is supported by notable interspecies differences: human and chimpanzee AREG share 98.0% amino acid identity, whereas human and mouse AREG share only 71.4% (5). Furthermore, the promoter region of AREG conservatively contains three TCF/LEF binding sites that are absent in rodents, suggesting that its regulatory mechanisms specialized later in mammalian evolution. At present, the expression and function of AREG during normal embryonic liver development and early postnatal stage remain unclear. Given the potential link between liver regeneration and developmental processes, analyzing the role of AREG in the physiological development may help elucidate the biological significance of the AREG-EGFR signaling axis across of the entire life cycle. Studies have shown that AREG plays a critical role in the development of mammary glands (6), oocytes (7), and bone tissue (8). More direct evidence comes from embryological research: the addition of AREG to porcine pre-implantation embryo culture specifically promotes the proliferation of trophoblast cells, thereby increasing the total cell number of blastocysts (9). This reveals the fundamental role of AREG in supporting early embryonic growth and development. Furthermore, large-scale expression profiling has confirmed that AREG is basally expressed in a variety of normal human tissues, including the liver. Collectively, these findings indicate that AREG serves as a crucial pro-proliferative signaling molecule during the developmental stage. This provides a developmental continuity explanation for the biological logic by which AREG is rapidly “recruited” in the adult liver following injury to initiate regenerative programs.

Among EGF-like peptides, AREG is unique due to a specific feature of its primary structure: a hydrophilic, highly positively charged N-terminal region rich in lysine and arginine residues, located adjacent to the C-terminal EGF domain. This structure dictates its receptor interaction pattern. In contrast to other EGF family members (such as BTC, EGF, and TGF-*α*), AREG binds to EGFR with lower affinity and reduced stability. Radioligand competition assays shown an IC₅₀ of 2.4 × 10^−8^ M for AREG, which is significantly higher than the 1.3 × 10^−10^ M observed for BTC. These binding characteristics consequently influence downstream events, resulting in distinct EGFR endocytosis and signaling kinetics compared with other ligands (10). Nevertheless, as a member of the EGF family, AREG can compete with EGF for binding to certain EGFRs. AREG activates fundamental intracellular signaling cascades that regulate cell metabolism, inflammation, and the cell cycle progression. Key downstream signaling pathways include the rat sarcoma-rapidly accelerated fibrosarcoma-MAPK/ERK kinase-extracellular signal-regulated kinases 1/2 (Ras–Raf–MEK-ERK1/2) pathway, which governs proliferation and differentiation; the signal transducer and activator of transcription 3 (STAT3) and signal transducer and activator of transcription 5 (STAT5) pathways, which also regulate these processes; and the phosphatidylinositol 3-kinase-protein kinase B-mechanistic target of rapamycin (PI3K-Akt–mTOR) pathway, which is crucial for cell survival (11). Extensive studies have collectively demonstrated that AREG, through binding to EGFR, plays a central role in tissue repair and fibrosis. In the context of acute injury and within epithelial tissues, AREG exerts a protective reparative function by promoting cellular proliferation and regeneration (12). Conversely, under chronic injury conditions—particularly in parenchymal organs—sustained high expression of AREG activates fibroblasts via the ADAM17-EGFR signaling axis, leading to excessive extracellular matrix (ECM) deposition and pathological fibrosis (13–15). This functional duality reflects the “dose–time–microenvironment” dependency of AREG activity, suggesting its potential as a stage-specific therapeutic target during disease progression. AREG exhibits significant expression heterogeneity across diverse hepatic pathologies, including hepatocellular carcinoma (HCC) progression, inflammatory microenvironment remodeling, biliary atresia (BA), and cholestatic injury. This review examines the known role of AREG in liver physiology and pathology, systematically summarizing its expression regulation, cellular sources, and downstream signaling pathways across diseases contexts, and discussing the challenges and translational prospects of targeting AREG.

## 2 The regulatory effect of AREG on liver regeneration

### 2.1 AREG orchestrates hepatocyte proliferation and liver regeneration following hepatectomy

The liver, the largest visceral organ in humans, possesses a regenerative capacity, exemplified by its ability to fully regenerate from as little as 25% of its residual volume following partial hepatectomy (PH). This process is fundamentally dependent on EGF-mediated paracrine signaling mechanisms. This extraordinary regenerative potential underscores the evolutionary conservation of EGF’s dominant regulatory role in hepatic homeostasis and tissue repair (16). Following PH, rapid changes in gene expression, receptor activation, and transcriptional factor activity occur in the remaining liver. Specifically, more than 100 genes in hepatocytes are upregulated within the first hour, reflecting a complex and robust regulatory response that explains the liver’s unique regenerative capacity compared with other organs (17). During liver regeneration, a precise dynamic balance is maintained between proregenerative and inhibitory regulatory factors.

The central regulatory mechanism of hepatocyte regeneration involves two receptor–ligand growth factor signal systems: the hepatocyte growth factor (HGF)—mesenchymal-epithelial transition factor receptor axis and epidermal growth factor receptor (EGFR) -ligand family system. Notably, EGFR was the first transmembrane receptor shown to be involved in liver regeneration. Researchers used RNA interference to EGFR expression in rat liver and observed impaired regenerative capacity, confirming that EGFR is a key regulator of liver regeneration. Studies in rodent models have demonstrated rapid activation of the EGFR pathway within 30 min after PH, whereas HGF signaling primarily drives hepatocyte proliferation during the early stages of regeneration, within 24 h after surgery (18).

EGF ligands associated with liver regeneration include EGF, AREG, transforming growth factor-*α* (TGF-α), and HB-EGF. Although these ligands differ in their mode of EGFR activation and downstream effects, their overall function is to promote hepatocyte proliferation. Recent studies comparing the efficacy of EGF ligand in small-for-size graft regeneration have identified AREG as a critical mediator. While AREG is undetectable in quiescent hepatic parenchyma under homeostatic conditions, PH induces robust hepatic expression of AREG through paracrine mediators, including Wilms’ tumor protein 1 (WT-1), prostaglandin E2 (PGE2), and interleukin-1β (IL-1β). Genetic ablation of AREG in murine models results in delayed liver regeneration, indicating its indispensable role in priming hepatocyte proliferation during the priming phase. Clinically, partial liver transplantation has become an important strategy to address global organ shortages. However, graft success largely depends on the graft-to-recipient size ratio, as excessively small graft volumes significantly increase the risk of posttransplant liver failure. These observations highlight the clinical relevance of AREG-mediated regenerative mechanisms (19, 20). Compared with normal-sized grafts, AREG levels remain low in small grafts and are insufficient to support regeneration. Neutralization of AREG impairs regeneration in normal grafts, whereas exogenous AREG supplementation enhances regeneration in small grafts, underscoring its essential role in hepatocyte proliferation. Although the activation of AREG is closely associated with a disintegrin and metalloproteinase 17 (ADAM17), studies have shown that ADAM17 expression remains stable during insufficient regeneration in undersized grafts, excluding it as a primary cause of regenerative failure (21, 22). This finding further emphasizes the unique contribution of AREG.

During the initiation of liver regeneration, AREG plays a “pioneer” signaling role. Its rapid and transient burst of expression within hours after injury “paves the way” and “sets the tone” for subsequent broader and more sustained regenerative programs (dominated by factors like EGF and HGF). Collectively, these studies establish the central role of EGFR signaling in hepatic regeneration. However, only the study by Liu et al. (21) directly and explicitly demonstrated the critical function of AREG. Using a mouse model of small-for-size liver transplantation, the research showed that exogenous AREG markedly enhanced graft regeneration by activating EGFR and its downstream signaling pathways. This finding provides a specific molecular mechanism and a potential therapeutic strategy for addressing poor regeneration in small grafts, a challenge previously highlighted by Sugawara et al. (20). The importance of AREG is further supported indirectly by other studies. Paranjpe et al. (19) demonstrated that EGFR inhibition severely compromises liver regeneration, underscoring the importance of its activating ligands including AREG. Similarly, Mao et al. (18) in their review, identified AREG as a key member of the EGF family involved in liver regeneration (Table 2).

**Table 2:** EGF ligands in liver regeneration.

Ligand	Source	Peak	Key role
AREG	Hepatocytes	Earliest	Primer: First signal to “wake up” hepatocytes.
HB-EGF	Sinusoidal cells	Very early	Initiator: Links blood flow to regeneration and protects cells.
EGF	Intestinal glands	Sustained	Basal signal: External supply; works best with other hormones.
TGF-α	Hepatocytes	Mid-phase	Amplifier: Autocrine loop to sustain proliferation.

### 2.2 AREG-mediated signaling pathways in hepatic regeneration

**Figure 1:**
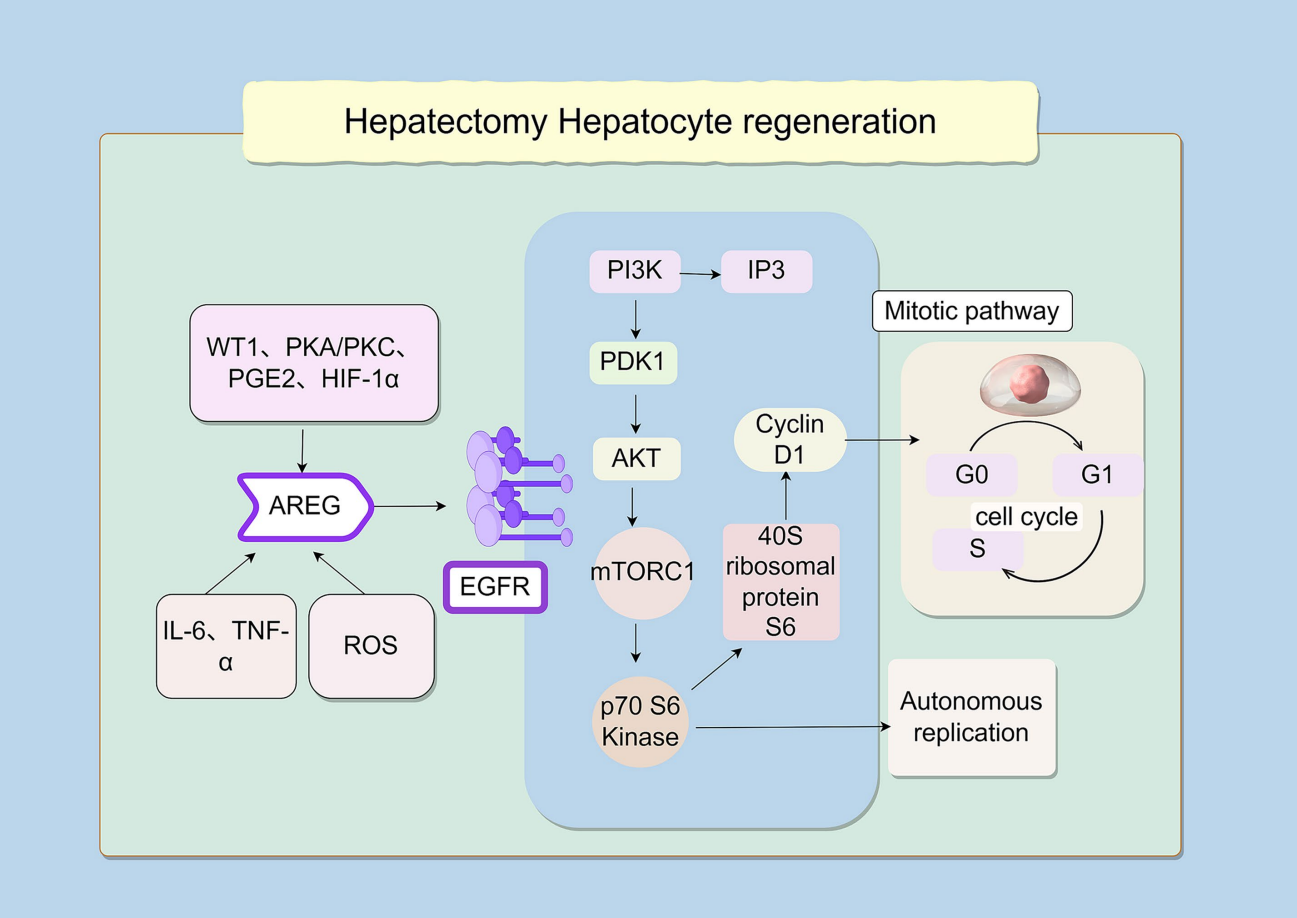
Signaling pathway of hepatocyte regeneration after partial hepatectomy. The illustration depicts the key signaling pathway driving hepatocyte regeneration. Upstream inducers including Wilms’ tumor protein 1 (WT1), protein kinase A/protein kinase C (PKA/PKC), prostaglandin E₂ (PGE_2_), and hypoxia-inducible factor 1*α* (HIF-1α), together with inflammatory cytokines such as interleukin-6 (IL-6), and reactive oxygen species (ROS) converge to stimulate amphiregulin (AREG) production. AREG binds to and activates epidermal growth factor receptor (EGFR). EGFR activation triggers the central phosphatidylinositol 3-kinase–phosphoinositide-dependent kinase 1–protein kinase B (PI3K-PDK1-AKT) signaling axis, leading to activation of mechanistic target of rapamycin complex 1 (mTORC1). Activated mTORC1 subsequently phosphorylates and activates p70 S6 kinase, which promotes protein synthesis through phosphorylation of the 40S ribosomal protein S6 and upregulates cyclin D1 expression. Collectively, these events drive the transition from the G0/G1 phase to S phase of the cell cycle, culminating in autonomous hepatocyte proliferation via the mitotic pathway.

The regulatory network controlling AREG biosynthesis during liver transplantation involves activation of Wilms’ tumor protein 1 (WT1), protein kinase A (PKA) and protein kinase C (PKC) signaling, prostaglandin E₂ (PGE_2_), and hypoxia-inducible factor 1α (HIF-1α). Elevated AREG promotes hepatic regeneration in size-constrained grafts by inducing EGFR-dependent phosphorylation of extracellular signal-regulated kinases 1/2 (ERK1/2) and protein kinase B (Akt). This signaling synergizes with mitogen-activated protein kinase (MAPK) pathway activation to drive compensatory hyperplasia. Mechanistically, AREG-EGFR engagement initiates phosphatidylinositol 3-kinase (PI3K)-mediated generation of phosphatidylinositol (3,4,5)-trisphosphate (PIP_3_), which recruits downstream effectors, including phosphoinositide-dependent kinase 1 (PDK1) and Akt. Deficiency of 3-Phosphoinositide-Dependent Protein Kinase 1 (PDK1) profoundly impairs post-hepatectomy liver regeneration, confirming its non-redundant role in this signaling axis (23). As noted by Mao et al. (18), EGFR influences hepatocyte proliferation through multiple downstream pathways, of which PI3K/Akt is only one. As a key effector of PI3K signaling, Akt regulates liver regeneration by activating mechanistic target of rapamycin (mTOR) and p70 S6 kinase, which in turn phosphorylate the40S ribosomal protein S6 to control protein synthesis and cell division. Cell cycle reprogramming strongly influences regenerative outcomes, with cytokine networks facilitating hepatocytes transition from G0 quiescence to G1 competence during the priming phase (24). Overall, the signaling network governing hepatocyte proliferation forms a complex regulatory hierarchy. Although EGFR ligands share receptor-binding specificity, they exhibit functional diversity across biological processes—including liver regeneration—through-ligand specific signaling outputs. With this complex signaling landscape, AREG displays a unique regulatory role that cannot be fully compensated by other EGFR ligands, particularly in establishing the epigenetic and metabolic conditions needed for regenerative capacity (25) (Figure 1).

### 2.3 Exploratory research opportunities

The precise mechanisms regulating AREG expression remain incompletely understood, particularly with respect to the cis-regulatory elements and the ways in which its downstream signals differ from that of other EGFR ligands. To advance toward clinical application, it is essential to validate the therapeutic effects of AREG in small-for-size graft models across multiple species. Achieving this goal will likely require developing targeted delivery strategies, such as hepatotropic adeno-associated virus vectors for gene therapy or liver-targeted fusion proteins. In addition, advanced experimental models, including organoids may prove instrumental in deciphering the complex regulatory networks involved in AREG-mediated regeneration. An additional unresolved issue is the AREG biosynthetic paradox: although a disintegrin and metalloproteinase 17 (ADAM17) is responsible for processing and activating pro-AREG, its expression remains stable during the inadequate regeneration in small-size grafts, whereas AREG levels are markedly reduced. This paradox highlights a critical knowledge gap and underscores the need for negative multi-omics approaches to map AREG’s context-dependent interactome.

## 3 Role of AREG in liver injury and fibrosis

### 3.1 AREG drives the progression of multiple types of acute and chronic hepatitis

Inflammatory mediators are essential pathogenic drivers of hepatic injury. In most human chronic liver diseases (CLD), both EGF and its cognate receptor are significantly upregulated, with accumulating evidence indicating their critical involvement in disease progression. Recent studies have demonstrated persistent overexpression of AREG during episodes of acute hepatic inflammation and acute liver injury, highlighting its potential regulatory role in acute-phase pathological processes (26). Measurement of ligand levels during acute liver inflammation have revealed a selective increase in AREG, but not in EGF, suggesting a distinct and potentially specialized role for AREG in the inflammatory response. Bacterial lipopolysaccharide (LPS) robustly induces hepatic AREG expression in murine models, an effect that is recapitulated in isolated hepatocytes stimulated with proinflammatory cytokines such as interleukin-1β (IL-1β), confirming cytokine-mediated AREG regulation. Notably, AREG expression is significantly increased in preclinical models of nonalcoholic fatty liver disease (NAFLD), where it drives the transcriptional activation of proinflammatory cytokines in hepatocytes. Mechanistically, AREG activates the nuclear factor κB (NF-κB) and MAPK signaling pathways, leading to upregulation of inducible nitric oxide synthase and cyclooxygenase-2 (COX-2), thereby enhancing the production of nitric oxide (NO) and prostaglandin E₂ (PGE_2_) (27, 28).

Recent mechanistic insights have uncovered an additional molecular dimension of AREG pathophysiology in NAFLD and nonalcoholic steatohepatitis (NASH). Pathologically elevated AREG levels, characteristic of NASH progression, directly impair mitochondrial bioenergetics by suppressing transcription of mitochondrial transcription factor A (TFAM), a central regulator of mitochondrial DNA replication and respiratory chain integrity. This AREG-TFAM regulatory axis disrupts mitochondrial cristae architecture, reduces oxidative phosphorylation capacity, and amplifies reactive oxygen species (ROS) production, thus establishing a self-perpetuating cycle of metabolic dysfunction and hepatocellular injury.

AREG has been characterized as a “crisis molecule,” rapidly induced via nan c-JUN/ Yes-Associated Protein (YAP)-mediated epigenetic reprogramming in response to intracellular stressors, such as mitochondrial damage, functioning as a cell-autonomous survival response under critical conditions (29, 30). The cellular identity secreting AREG is a core factor in predicting its functional outcome. AREG from different sources contributes to constructing distinct pathological niches. Emerging evidence further indicates a pivotal role of AREG secreted by regulatory T cells (Tregs), in coordinating hepatic fibrogenesis and NASH-associated glucose metabolic dysregulation. Treg-derived AREG acts as a multifunctional mediator that links immune modulation with metabolic reprogramming, thereby amplifying pathological crosstalk within the fibrotic hepatic niche (31). Clinical and pathological analyses have demonstrated a significant positive correlation between intrahepatic Treg abundance and AREG expression levels within injury microenvironments (32). Additional studies indicate that Treg-derived AREG activates hepatic stellate cells (HSCs) via the EGFR signaling cascade, promoting insulin resistance and glucose intolerance in a NASH-dependent manner.

Collectively, these findings presents a novel perspective AEEG function, suggesting a crucial role in maintaining liver homeostasis. This view contrasts with earlier interpretations by Sheedfar et al. (33), who emphasized AREG’s involvement in metabolic stress, and the foundational work of Michalopoulos et al. (17), which highlighted its regenerative function. These apparent discrepancy may arise from differences in detection sensitivity or from cell-specific patterns of AREG expression. We propose that AREG functions as an “inducible reserve factor,” remaining at very low levels under strict quiescence conditions but capable of rapid upregulation in response to subtle physiological or stress signals. Consequently, varying definitions of the “normal state” may contribute to divergent experimental observations. Future studies employing single-cell technologies will be essential to precisely quantify AREG expression across clearly defined physiological and pathological contexts.

### 3.2 AREG plays a role in the majority of fibrotic conditions

Sustained hepatic inflammation is a prerequisite for fibrotic progression, with compelling evidence showing that AREG and inflammatory mediators cooperatively drive hepatocellular injury and fibroinflammatory responses. This interplay mechanistically pathological cascades, critically contributing to the initiation and perpetuation of fibrogenesis (34). Evidence from diverse animal models of hepatitis-associated hepatic fibrosis consistently demonstrates upregulation of AREG in both murine and human HSCs. Elevated AREG expression correlates positively with profibrotic biomarkers, including transforming growth factor-β1 (TGF-β1) and phosphorylation of Mothers Against Decapentaplegic Homolog 2/3 (Smad2/3) (35). In both murine and human ECM-producing cells, AREG expression is strongly induced via platelet-derived growth factor (PDGF)-mediated EGFR transactivation. Acting as a pivotal survival factor for ECM-generating cells, AREG sustains fibrotic matrix deposition *in vivo* by coordinating the activation of multilayered anti-apoptotic pathways, thereby preserving the viability of fibrogenic cells during progressive fibrogenesis (36). Beyond direct activation of ECM-producing cells, AREG exerts pleiotropic fibrogenic effects by upregulating tissue inhibitor of metalloproteinases 1 (TIMP1) and connective tissue growth factor (CTGF) through intercellular crosstalk. AREG derived from hepatocytes and Kupffer cells is highly responsive to inflammatory mediators, amplifying fibrogenic responses via autocrine and paracrine signaling. This mechanism propagates profibrotic signaling gradients, fueling the self-sustaining progression of hepatic fibrosis (37).

Multiple clinical and preclinical studies consistently identify AREG as a key driver of liver fibrosis. Kim et al. (38) first observed significantly upregulated AREG expression in liver tissues from patients with liver cirrhosis of various etiologies, including metabolic dysfunction-related steatohepatitis, viral hepatitis, and alcoholic liver disease, indicating a broad association with the progression of CLD. Wang et al. (39) confirmed this findings in metabolic dysfunction-related steatohepatitis (MASH) patients, showing that hepatic AREG mRNA level was approximately sevenfold higher than in controls. Moreover, AREG expression positively correlated with HSCs activation and overall fibrosis stage, establishing a direct link between AREG levels and fibrosis severity. Mechanistic evidence from Fujiwara et al. (40), further demonstrated that the proteasome inhibitor carfilzomib alleviate liver fibrosis by specifically suppressing AREG expression in HSCs, highlighting AREG’s central role in HSC activation and its potential as a antifibrotic therapeutic target. Collectively, from population studies to mechanism intervention, current evidence strongly supports AREG as a promising and actionable target for antifibrotic therapy.

AREG also plays a critical role in neonatal fibrous inflammatory cholangiopathy BA. In this condition, liver-resident mucosal associated invariant T (MAIT) cells significantly upregulate AREG expression via a T cell receptor (TCR)-dependent activation. Paracrine AREG from MAIT cells drives the fibrotic phenotypic transition of cholangiocytes (41). In addition to promoting differentiation, MAIT cell- stimulates proliferation, amplifying multicellular inflammatory cascades and fibrotic processes in of bile duct epithelial cells (42).

In summary, although studies by Savage and Xiao examined distinct diseases, they collectively highlighted the context-dependent role of AREG in liver pathology. In NASH model, Treg-derived AREG primarily targets HSCs, promoting a profibrotic phenotype and exacerbating tissue injury and scar formation (31). In contrast, in BA, MAIT cell-derived AREG preferentially targets cholangiocytes, stimulating their proliferation and driving a reparative ductular reaction (42). These contrast underscores that AREG ultimate biological effect is determined not by the molecule itself, but by its cellular source, target cell type, and local disease microenvironment. Consequently, future therapeutic strategies should adopt a precision-intervention approach, clearly defining whether AREG acts as a “disruptor” or a “repairer” at disease stage. Such a nuanced strategy is particularly essential to avoid impairing AREG’s critical reparative while blocking its pathogenic activity.

### 3.3 Protective effect of AREG on liver injury

AREG exhibits a paradoxical dual function in hepatic pathophysiology: while it can exacerbate disease progression through proinflammatory and pro-fibrotic effects, it also exerts hepatoprotective properties by regulating cellular survival and regenerative mechanisms (43). Studies using the concanavalin A (Con A)-induced acute immune-mediated liver injury model highlight AREG’s therapeutic potential in autoimmune hepatitis. Specifically, AREG administration mitigates hepatocellular injury through a dual apoptotic mechanism: it enhances anti-apoptotic pathways by upregulating B-cell lymphoma-2 (Bcl-2) and Bcl-2 extra-long (Bcl-xL), while simultaneously suppressing pro-apoptotic signaling by inhibiting caspase-3 activation. This coordinated regulation significantly improves hepatic functional parameters. Furthermore, AREG enhances Interleukin-22 (IL-22) production in hepatic CD3^+^ T cells by activating the IL-22/STAT3 signaling axis, inducing anti-inflammatory mediators, proliferative genes, and antioxidant defenses. Multi-omics analyses confirm that paracrine immunomodulation drives hepatocyte regeneration and architectural restoration in injured livers (44). In a mouse model of Fas agonist-induced fulminant liver injury, AREG expression is significantly upregulated, and its protective effect is mediated through activation of the Akt/STAT3 survival signaling pathway (45). Notably, AREG immunomodulatory function is cell type-specific. In immune-mediated hepatitis, it mitigates excessive immune responses by modulating the functional status of liver-resident ST2 + regulatory T cells (Tregs) and Group 2 innate lymphoid cells (ILC2s), while preserving tissue repair capacity (46). The regulatory network of AREG is complex: endogenous interleukin-33 (IL-33) released by injured hepatocytes can promote fibrosis by activating the AREG/TGF-β1 axis via the ST2 receptor, exogenous IL-33 exerts anti-inflammatory effects by inducing ILC2 secretion of AREG. This paradox suggests that AREG’s function is dictated by the spatiotemporal dynamics of microenvironmental factors (47).

Under acute inflammatory stress, AREG activates the EGFR-AKT/ERK1/2 signaling axis to counteract TNF-*α*-mediated hepatocyte apoptosis, exerting a direct hepatoprotective effect (48). This aligns with Zhou et al. (49), who showed that hepatocyte-derived AREG induces HSCs apoptosis via a STAT1-dependent pathway, contributing to protective regulation in cirrhosis. Together, these findings suggest that the AREG’s protective role is cell source–specific: hepatocyte-derived AREG primarily mediates anti-apoptotic and proregenerative effects, whereas HSC-derived AREG may exert protective functions under specific conditions. For example, Zou et al. (50) reported in patients with metabolic dysfunction–associated fatty liver disease, overall hepatic AREG is decreased, while HSC-derived AREG retains protective activity. These observations indicates that the biological outcome of AREG signaling depends on expression level, cellular origin, and disease stage. In acute or early-stage injury, hepatocyte-derived AREG likely supports protection through anti-apoptotic and proregenerative mechanisms. In contrast, under conditions of chronic injury, sustained high expression of AREG from HSCs or other sources may drive pathological fibrotic progression.

Mechanistic diversity further characterizes AREG-mediated protection. Beraza et al. (45) and Wu et al. (44) confirmed the hepatoprotective role through distinct pathways—EGFR-dependent hepatocyte proliferation and IL-22 induction, respectively. Wachtersdorf et al. (47) highlighted the importance of ST2^+^ Treg-derived AREG in suppressing immune-mediated hepatitis and maintaining homeostasis. Such heterogeneity supports Eckstein et al. (51) view that AREG function is shaped by the nature of injury and the surrounding cytokine milieu, positioning AREG as a multifunctional modulator within broader inflammatory networks.

Overall, AREG’s impact in liver pathology is fundamentally dualistic, acting as a determinant of tissue restoration or pathological fibrosis depending on the cellular target and disease stage. In acute liver injury, hepatocyte-derived AREG predominantly functions as a hepatoprotective factor by upregulating anti-apoptotic mediators (Bcl-2, Bcl-xL) and inhibiting caspase-3 activation, preserving parenchymal integrity and facilitating compensatory mitosis. However, during chronic inflammation, sustained AREG expression—particularly from HSCs—shifts its primary target toward fibrogenesis. In this context, AREG induces HSCs to transition into myofibroblasts and upregulates fibrogenic factors such as TIMP1 and CTGF, disrupting ECM homeostasis. Thus, AREG should not be simplistically classified as profibrotic or protective. Instead, it is a dynamically regulated molecule influenced by cellular source, expression level, and disease context. Future studies should focus on elucidating the spatiotemporal expression patterns of AREG from different cellular origins and clarifying stage-specific signaling networks. Such detailed mapping is essential for developing targeted therapeutic strategies that mitigate fibrosis while preserving regenerative potential.

### 3.4 AREG orchestrates viral persistence and hepatic injury

Hepatitis C virus (HCV) infection significantly upregulate the expression of AREG in hepatocytes. Mechanistic studies reveal that AREG plays a critical role in efficient assembly and release of HCV virions. HCV drives sustained AREG overexpression through a three-step mechanism. First, double-stranded RNA generated during viral replication is recognized by receptors, such as RIG-I, activating NF-κB and initiating the transcription of AREG. Next, the viral NS3 protease amplifies this signal by activating the MAPK/ERK pathway, enabling the transcription factor AP-1 to synergize with NF-κB, thereby robustly enhancing AREG transcription. Finally, secreted AREG binds to EGFR on hepatocytes, activating downstream pathways such as RAS–RAF–MEK–ERK. This establishes an autocrine positive feedback loop that maintains persistently high AREG expression, creating a favorable environment for viral proliferation (52). Building upon AREG’s role in cellular growth regulation, researchers propose an apoptosis-mediated pathogenesis model: AREG may facilitate persistent HCV infection by suppressing apoptosis in infected hepatocytes. This survival advantage allows prolonged viral persistence, ultimately accelerating the progression from chronic hepatitis to advanced liver cirrhosis and HCC (53). Additionally, as a secreted protein, AREG participates in remodeling the tumor microenvironment by modulating intercellular communication and mediating paracrine signaling between infected hepatocytes/HCC cells and non-infected stromal cells. In the early stages of HCV-induced liver pathogenesis, HCV stimulates inflammatory responses, fibrotic processes, and angiogenesis-related factors via EGFR-ERK signaling cascade. Reprogramming of this multidimensional microenvironment establishes a protumorigenic niche, providing a critical pathological foundation for malignant transformation and driving HCC initiation and progression (54).

Studies using a murine model of hepatitis B virus (HBV) infection have identified a distinct subset of hepatic macrophages specifically express AREG. This subpopulation enhances the immunosuppressive activity of Tregs by upregulating inhibitory molecules, including cytotoxic T-lymphocyte-associated protein 4 (CTLA-4), inducible T cell co-stimulator, and cluster of differentiation 39 (CD39), thereby impairing CD8 + T cell function. These findings reveal a novel mechanism by which HBV evades host immune surveillance.

AREG also exerts hepatoprotective functions, linking bile acid metabolism to cellular defense. Acutely, it mitigates hepatocyte apoptosis and necrosis by activating the EGFR signaling pathway to counteract bile acid toxicity. Chronically, it suppresses bile acid synthesis by inhibiting Cytochrome P450 Family 7 Subfamily A Member 1 (CYP7A1) expression, reducing the accumulation of toxic bile acids at the source. This dual mechanism highlights AREG’s potential as a therapeutic target for cholestatic liver diseases (55). Emerging evidence also implicates AREG in other hepatic processes, including mitigating erythropoietic protoporphyria (EPP)-related liver complications and coordinating with estrogen to regulate ROS metabolism in hepatocytes (56, 57).

**Figure 2:**
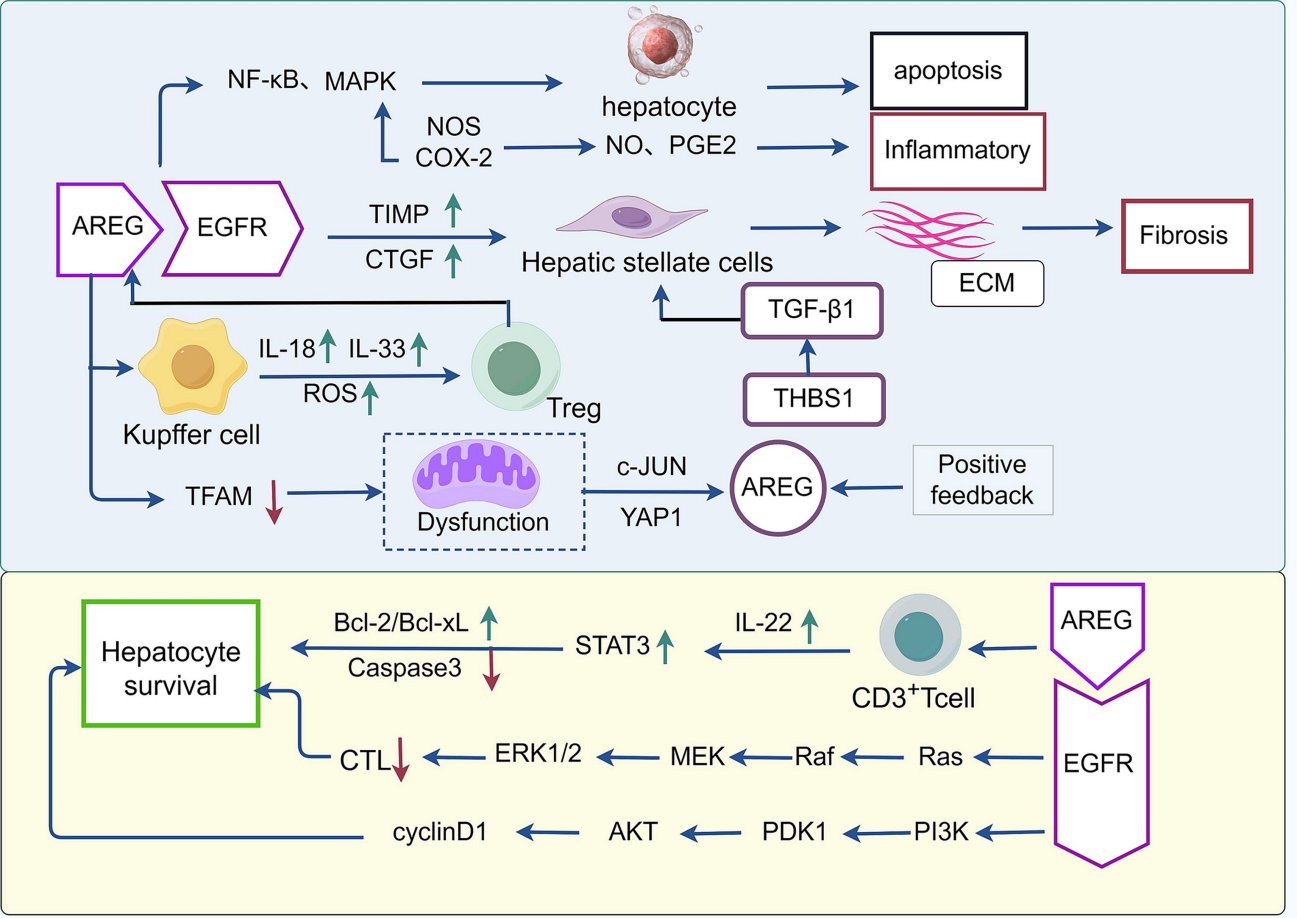
The regulatory pathway of AREG in hepatitis and liver fibrosis. In nonalcoholic fatty liver disease (NAFLD), amphiregulin AREG significantly induces the expression of inducible nitric oxide synthase (NOS) and cyclooxygenase-2 (COX-2), increasing the secretion of nitric oxide (NO) and prostaglandin E_₂_ (PGE_2_) through NF-κB and MAPK signaling pathways, thereby accelerating hepatocyte apoptosis and the inflammatory response. In liver fibrosis, AREG upregulates tissue inhibitor of metalloproteinases1 (TIMP1) and connective tissue growth factor (CTGF). Interleukin-18 (IL-18) and interleukin-33 (IL-33) activate the STAT5/ERK signaling pathway to specifically promote AREG biosynthesis in regulatory T (Treg) cells, which synergistically stimulates hepatic stellate cells (HSCs) to enhance fibrosis. Loss of mitochondrial transcription factor A (TFAM) induces mitochondrial stress, AREG transcription via c- Jun N-terminal kinase (JNK) and Yes-associated protein (YAP), and, in coordination with thrombospondin-1 (THBS1), directly activates HSCs through Smad2/3 phosphorylation, accelerating extracellular matrix deposition. In immune-mediated hepatitis, AREG upregulates Bcl-2 and Bcl-xL while inhibiting caspase-3. It also induces IL-22 secretion by hepatic CD3^+^ T cells activating the IL-22/STAT3 signaling pathway to promote anti-inflammatory mediators, cell proliferation–related genes, and antioxidant proteins, thereby facilitating liver tissue repair and regeneration. Furthermore, AREG binds to EGFR on hepatocytes, activating downstream RAS–RAF–MEK–ERK pathways. This establishes an autocrine positive feedback loop that maintains high AREG expression, creating a favorable environment for viral proliferation.

AREG is not simply a “good” or “bad” molecule; its function is highly context-dependent. The key determinants steering it toward regeneration or pathology lie in the pathological stage, cellular source, and microenvironment in which it acts. Collectively, these studies reveal the pleiotropic effects of AREG in liver diseases, but significant contradictions exist regarding its direction of action. The most fundamental contradiction lies in its dual role in viral liver diseases: in HCV models, it promotes virus survival and inflammation, whereas in cholestatic models, it inhibits damage. This contradiction, determined by disease type, suggests that AREG may not merely be an effector molecule executing repair but rather an early-stage switch regulated by the “instruction” of injury nature, determining the liver’s overall response mode. We propose a hypothetical framework: when injury originates from a threat perceived by the immune system (e.g., HCV), AREG derived from immune cells tends to initiate a response centered on “immune regulation,” potentially at the cost of suppressing necessary anti-pathogen responses. Conversely, when injury stems from direct physico-chemical toxicity (e.g., bile acids, drugs), AREG derived from epithelial cells initiates a response focused on “epithelial repair,” which is efficient but localized. This framework positions AREG at an upstream integration node of the injury signaling network, where its “double-edged sword” effect essentially represents the “hijacking” or “misdirection” of the host repair program by different etiologies. Consequently, the future research focus should shift from “whether AREG promotes disease or protection” to “which upstream signals determine the secretory cells and functional repertoire of AREG” and “how to precisely modulate this switch to steer it toward purely protective repair.” This discrepancy likely arises from the interaction between disease type, cellular source, and microenvironment signals, suggesting that AREG function as a “double-edged sword” in liver diseases. Its role must therefore be analyzed precisely within the specific pathological context (Figure 2).

## 4 The critical role of AREG in HCC

### 4.1 The AREG-EGFR pathway promotes the survival of HCC cells

The pivotal role of EGFR-mediated signaling in HCC pathogenesis is widely recognized and forms the basis for most clinically approved targeted therapies against this malignancy (58). EGFR overexpression occurs in approximately 68% of human HCC cases, with its activation strongly correlating with tumor aggressiveness, metastatic potential, and reduced patient survival. AREG promotes hepatocyte proliferation and drug resistance primarily via activation of the EGFR-ERK pathway. Although AREG expression has been associated with favorable prognostic markers such as lower Edmondson grade and reduced alpha-fetoprotein (AFP) levels, this correlation may reflect a distinct molecular subtype rather than a protective effect.

In HCC progression, AREG drives tumor advancement by establishing a self-sustaining autocrine loop though persistent EGFR-ERK activation (59). This loop supports dual metabolic reprogramming, characterized by enhanced glycolysis and metabolic plasticity, forming a core self-reinforcing circuit that fuels tumorigenesis (60). This loop is further amplified by dysregulation of the Hippo tumor-suppressor pathway: nuclear accumulation of YAP, in complex with the TEA domain transcription factor (TEAD), directly targets the *AREG* gene, cementing persistent signaling (61, 62). AREG integrates multiple carcinogenic pathways, including EGFR to Hippo and Wnt/*β*-catenin—positioning it as a central hub rather than a solitary driver in HCC signaling network (63).

Additionally, AREG-driven network exhibits remarkable crosstalk with other key pathways. For example, the fibroblast growth factor 19 (FGF19)- fibroblast growth factor receptor 4 (FGFR4) axis converges on β-catenin stabilization and transcriptional activity at the AREG promoter (64). Together, these signals drives a pathological triad: metabolic reprogramming toward glycolysis (the Warburg effect), persistent oxidative stress from mitochondrial dysfunction, and sustained proliferative signaling, creating a self-perpetuating oncogenic cycle. AREG further promotes oncogenic plasticity by modulating RNA processing, including the downregulation of the splicing factor Synthetic lethal with U5 snRNA 7 (Slu7), which favors the production of the ΔEx2p73 oncogenic isoform (65). Collectively, these findings underscores AREG as more than a growth factor —it functions as a central orchestrator of a pathological ecosystem that drives HCC progression and therapeutic resistance.

Despite extensive evidence establishing AREG as a key oncogenic driver (66, 67), its function is increasingly recognized as complex. For example, AREG expression induced by Hippo-YAP signaling extends beyond direct tumor promotion to include significant paracrine modulation of stromal cells (62). The limited clinical efficacy for EGFR inhibitors in HCC (58, 68) further suggests that AREG operates a highly redundant signaling network; and inhibiting AREG alone may be insufficient to halt disease progression. This highlights the “necessary but not sufficient” nature of AREG in hepatocarcinogenesis: it is indispensable for tumor maintenance, yet its full oncogenic potential requires cooperation with alternative pathways. Consequently, therapeutic strategies targeting the AREG-EGFR axis in isolation may face intrinsic limitations.

### 4.2 AREG: a central therapeutic target regulating chemotherapy resistance and the immune microenvironment in HCC

AREG critically mediates chemoresistance in HCC, with its suppression sensitizing tumor cells to both chemotherapy and TGF-*β*-induced apoptosis. Sorafenib is a first-line therapy for advanced HCC, and clinically, elevated serum AREG levels correlate with acquired sorafenib resistance, suggesting the potential benefits of combination therapies with EGFR inhibitors (69, 70). Importantly, this resistance was effectively reversed in preclinical models by coadministering either ERK1/2 or AHR inhibitors, highlighting a promising combination strategy to overcome therapeutic resistance (71). Piffkó et al. (72) demonstrated that while radiotherapy effectively kills local tumor cells, it concurrently induces secretion of high levels of AREG, paradoxically promoting distal metastasis. The mechanism involves AREG reprogramming myeloid-derived immune cells into an immunosuppressive state while upregulating the “do not-eat-me” signal CD47 on tumor cells. This dual pathway facilitates immune evasion and colonization of metastatic lesions. Combining radiotherapy with an AREG-blocking antibody effectively counteracts this prometastatic effect, suggesting a novel combinatorial approach to improve clinical outcomes. AREG expression in T cell subsets also modulates immune regulation within the tumor microenvironment, potentially influencing HCC immune evasion and insights from SLE-related immune dysregulation may illuminate broader cancer-immune interactions (73).

Beyond direct tumor effects, AREG remodels the tumor microenvironment via senescent stromal cells. These cells secrete AREG in a self-reinforcing loop that promotes stromal senescence, leading to dual oncogenic outcomes: enhanced tumor malignancy and therapy resistance, and paracrine induction of programmed cell death-ligand 1 (PD-L1) fostering immunosuppressive niches (74). Given its central role in these multidimensional mechanisms, AREG represents both a promising diagnostic biomarker and a potential therapeutic target for the HCC. Additional regulators of AREG expression include WT1, protein kinases A/C, PGE_2_, and HIF-1α, which activate downstream oncogenic signaling via ERK-1/2 and Akt phosphorylation. These findings position AREG as a key regulator of HCC progression, directly controlling proliferation-apoptosis balance and indirectly reshaping the tumor microenvironment. Consequently, targeting the AREG signaling network offers a promising therapeutic strategy for liver cancer.

**Figure 3:**
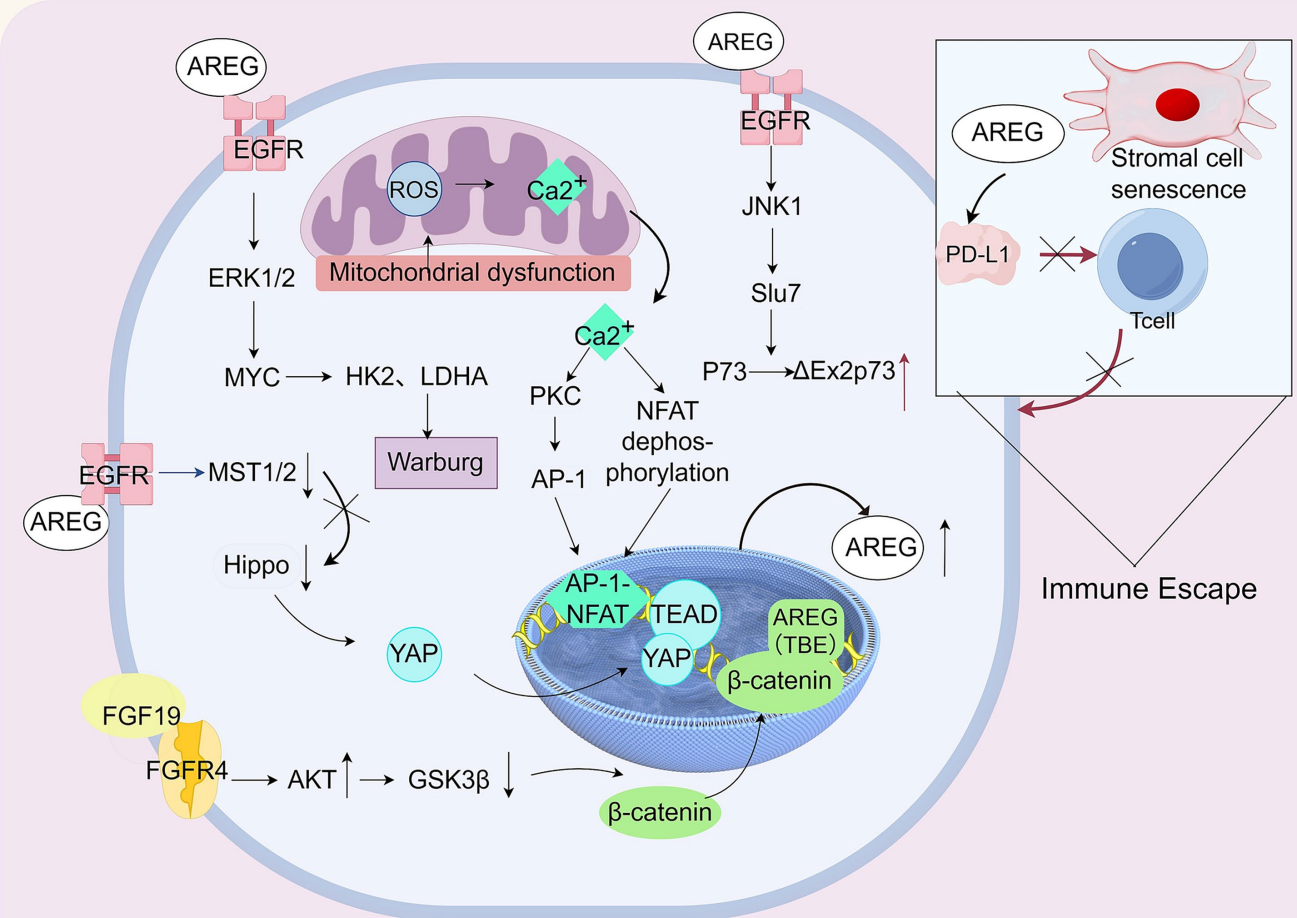
AREG promotes the proliferation of liver cancer cells through multiple pathways. The binding of AREG to its cell membrane receptor, EGFR, initiates a signaling cascade that activates key regulatory proteins, including YAP and β-catenin. This activation influences cell fate decisions and interacts with pathological states such as mitochondrial dysfunction and hypoxia, contributing to a self-perpetuating vicious cycle. AREG -EGFR engagement triggers, initiating a complex signaling cascade in profound cellular reprogramming. This activation stimulates the MAPK pathway, notably ERK1/2, leading to mitochondrial dysfunction and the release of reactive oxygen species (ROS) and calcium ions (Ca^2+^) into the cytosol. The liberated Ca^2+^ acts as a critical second messenger, activating PKC and the nuclear factors NFAT and AP-1, which drive *AREG* gene expression, establishing a potent positive feedback loop. Concurrently, AREG-EGFR signaling inhibits the Hippo pathway by inactivating MST1/2, stabilizing YAP and promoting nuclear translocation. Nuclear YAP partners with TEAD to directly activate the AREG promoter, further amplifying the signal. In a complementary pathway, FGF19-FGFR4 signaling inhibits GSK-3β via Ser9 phosphorylation, preventing β-catenin degradation and promoting its nuclear accumulation. There, upon binding to its receptor FGFR4, FGF19 signaling inhibits the activity of GSK-3β through phosphorylation at its Ser9 residue. This inhibition prevents the degradation of β-catenin and promotes its nuclear translocation. Inside the nucleus, β-catenin forms a complex with transcription factors, which binds to specific TBEs within the AREG promoter, thereby directly driving AREG transcription. The integration of these signals orchestrates pro-tumorigenic processes, including a vicious cycle where mitochondrial dysfunction, ROS production, and the Warburg effect mutually reinforce each other. This self-sustaining loop, combined with Hippo pathway dysregulation, perpetuate aberrant cellular states and continuous AREG expression. Furthermore, AREG promotes oncogenic splicing via the EGFR/JNK1 axis by downregulating the splicing factor Slu7, favoring the production of the ΔEx2p73 oncogene. In therapy resistance, sorafenib can paradoxically upregulate AREG, which induces PD-L1 expression, fostering an immunosuppressive microenvironment that facilitates evasion from treatment.

Future research aimed at the precise intervention of this network may yield innovative, multipathway synergistic treatment paradigms. However, translating these insights into effective therapies faces challenges. The pleiotropic nature of AREG raises concerns about potential on-target toxicity. Furthermore, identifying reliable biomarkers to stratify patients most likely to benefit from AREG-targeted interventions remains a critical unmet need (Figure 3).

### 4.3 Association between AREG and specific types of liver cancer

Cholangiocarcinoma (CCA), the most lethal form of liver cancer accounting for 10–20% of primary hepatic malignancies, exhibits differential AREG expression between malignant and normal biliary epithelia. Clinically, elevated AREG levels correlate with aggressive tumor behavior and poor prognosis (75). Mechanistic studies reveal PGE-mediated upregulation of AREG via the cyclic adenosine monophosphate (cAMP)/PKA/cAMP response element-binding protein (CREB) signaling axis, directly driving proliferation of CCLP1 cholangiocarcinoma. Concurrently, AREG promotes angiogenesis in CCA by stimulating endothelial cell proliferation, migration, and vascular lumen remodeling, thereby establishing a tumor-supportive vasculature. Intriguingly, genetic deletion of Cullin3 (Cul3)—a critical component of the E3 ubiquitin ligase complex—triggers cholangiocarcinogenesis through Nrf2-mediated AREG overproduction. This paracrine signaling cascade induces hepatic inflammation while simultaneously reprogramming the tumor microenvironment *(TME)* via suppression of cytotoxic immune responses, ultimately driving CCA progression (76). Conventional understanding has largely viewed AREG as an EGFR ligand that drives autonomous proliferation in HCC. However, its role in CCA reveals a more complex narrative: AREG functions as a “master orchestrator” systematically reshapes the tumor-immune ecosystem. Genetic loss of Cul3 triggers aberrant AREG overexpression through Nrf2 stabilization—a process that ignites inflammation and, concurrently diminishes the tumor-immune defenses. This systemic remodeling positions AREG as a highly attractive therapeutic target. Notably, AREG overexpression is also driven by key oncogenes, such as YAP1, which directly promotes the invasive phenotype of CCA. Based on this core mechanism, preclinical studies have validated direct targeting of AREG using neutralizing antibodies, demonstrating antitumor efficacy comparable to the approved drug regorafenib. This provides direct evidence for translating AREG from a “mechanistic hub” into a viable “therapeutic target” (77). Fibrolamellar carcinoma (FLC), a rare hepatic malignancy predominantly affecting adolescents and young adults without underlying liver disease (78), shares molecular parallels with CCA. Mutant YAP1 in FLC transcriptionally activates oncogenic targets, including AREG and Collagen Type I Alpha 1 Chain (COL1A1), synergistically promoting tumor cell proliferation and fibrotic stromal formation (79, 80).

Beyond primary liver cancers, AREG functions as a key promoter of colorectal cancer liver metastasis. Gene expression profiling (e.g., microarray and RT-PCR) consistently reveals elevated AREG levels in primary tumors that metastasize to the liver. Its prometastatic role is mediated through EGFR downstream signaling, facilitating tumor cell invasion and survival, often in concert with ligands such as EREG. Multivariate analyses validate high AREG expression as an independent prognostic factor for increased metastatic risk and worse overall survival, highlighting its utility as a biomarker for risk stratification (81). A 2025 study in Nature (82) identified a previously unknown function of AREG within the intestinal tract. Activation of the cytochrome P450 family 27 subfamily A member 1 (CYP27A1)/ liver X receptor (LXR) pathway in gut epithelial cells induces AREG expression, which coordinately drives tissue repair and protective antitumor immunity dependent on B cells and CD8^+^ T cells. Although this study was conducted in an intestinal context, it reveals a fundamental mechanism: the LXR metabolic pathway, via AREG, links tissue healing to immune surveillance. This insight offers a valuable new framework for investigating AREG’s role in the immune microenvironment of the liver.

## 5 Conclusion and outlook

AREG exhibits a complex functional duality in liver pathology, with its specific role critically dependent on disease progression stage, cell lineage, and local microenvironmental cues (Table 3). Although extensive research has elucidated the pleiotropic regulatory effects of AREG on liver regeneration, fibrosis, and hepatocarcinogenesis, the precise dynamic switches that convert AREG from a “protective factor” into a “pro-pathological driver” remain elusive. Future investigative efforts must transcend the traditional paradigm of analyzing single molecules or isolated pathways and instead focus on integrated approaches: Evidence from single-cell multi-omics dynamic analyses highlights the distinct roles AREG plays in hepatocytes, stellate cells, and immune cell lineages. These insights further reveal an intricate interaction network involving AREG and pivotal pathways such as TGF-*β*1 and Wnt/β-catenin, with a particular emphasis on epigenetic regulation mediated by non-canonical EGFR signaling. Consequently, it is imperative to leverage advanced technologies, such as spatial transcriptomics, to construct a dynamic regulatory network of AREG within the liver microenvironment, clarifying how it mediates aberrant intercellular communication between key cell populations and jointly drivers malignant transformation. 2. Existing studies have confirmed that AREG, as an EGFR ligand, can be specifically packaged and enriched in exosomes which act as delivery vehicles capable of precisely transporting AREG to target cells and activating a series of key biological processes. For instance, exosomes derived from multiple myeloma cells (83), human colorectal cancer cells (HCA-7) (80), and non–small cell lung cancer cell lines (CRL-2868) (81) are rich in AREG. Future research should aim to develop a multi-group biomarker model based on exosomal AREG, explore neutralizing antibodies and gene-editing techniques targeting AREG, investigate their synergistic effects with immunotherapy or antifibrotic agents, and evaluate heterogeneity in therapeutic efficacy across different liver subtypes, such as viral and metabolic HCC. 3. AREG is associated with multiple pathological processes involving remodeling of the hepatic sinusoidal microenvironment, including activation of HSCs, promotion of endothelial cell proliferation, and transformation of macrophage phenotypes. We can reasonably believe that AREG is a factor related to the remodeling of the hepatic sinusoidal microenvironment. Addressing unresolved issues—such as the molecular switching of AREG functional polarity, its interaction with hepatic sinusoidal microenvironment remodeling, and the dynamic validation of AREG expression profiles across diverse liver diseases—will require patient-derived organoids. These models can simulate human-specific pathological processes, predict intervention targets, and help overcome safety challenges, including off-target toxicity. However, several challenges remain. Because AREG functions alongside other ligands such as Epiregulin and HB-EGF, specific inhibition may trigger compensatory upregulation of these counterparts. In addition, long-term inhibition of AREG during liver cancer therapy may impair the liver’s resilience to acute injury, whether from drug toxicity or postsurgical recovery—a concern particularly acute in HCC patients with underlying cirrhosis. Consequently, the major challenge lies in balancing effective tumor control with the preservation of physiological hepatic reserve. This precision strategies that selectively disrupt AREG’s oncogenic signaling while maintaining its regenerative functions in normal tissue are essential. This functional overlap complicates the success of single-target strategies. Furthermore, the role of AREG in modulating the immune landscape cannot be overlooked. Ultimately, integrating multidimensional research framework encompassing spatiotemporal dynamics and intercellular interactions, will be critical for advancing AREG from mechanistic investigation toward precision therapy, providing potential breakthrough strategies for major liver diseases including liver fibrosis and liver cancer.

**Table 3:** The role of AREG in liver disease.

Disease	Effect	Effector factors
Hepatectomy/post-liver transplantation	Promotes hepatocyte regeneration	PI3K, IP3, and AKT
NAFLD/NASH	Promotes inflammatory response	NF-κB, MAPK
Autoimmune hepatitis	Promotes liver tissue repair and regeneration	Bcl-2, Bcl-xL, Caspase-3, Akt, STAT3
Liver fibrosis	Accelerates liver fibrosis	TIMP1, CTGF, THBS1, TGF-β, Smad2/3
Viral hepatitis	Inhibit apoptosis of virus-infected cells	EGFR-ERK CTLA-4, ICOS, CD39
HCC	Promotes cancer cell proliferation and invasion	EGFR-ERK, Akt, β-catenin EGFR-JNK1
Cholangiocarcinoma	Promotes cholangiocarcinoma cell survival	cAMP/PKA/CREB
Fibrolamellar carcinoma	Promotes cancer cell proliferation and fibrotic matrix formation	YAP
